# The Hospital. Nursing Section

**Published:** 1903-07-18

**Authors:** 


### The Hospital.

### Nursing Section.

Contributions for this Section of "THE HOSPITAL" should be addressed to the EDITOR, "THE HOSPITAL" NURSING SECTION, 28 & 29 Southampton Street, Strand, London, W.C.

No. 877.—VOL. XXXIV. *SATURDAY, JULY* 18, 1903.

### Notes on News from the Nursing World.

### THE KING'S VISIT TO THE EASTBOURNE HOSPITAL.

ON Monday morning at half-past nine the King paid a visit to the Princess Alice Memorial Hospital, Eastbourne, and was taken over the institution by the matron, Miss M. Ramsay. His Majesty went into all the wards, spoke to every patient, and made inquiries as to their condition. He next visited the convalescent rooms, built in memory of the late treasurer, Mr. Gurney, and the nurses' sitting-room, with which he expressed himself charmed. In the matron's room the King wrote the following in the visitors' book:—"I paid a surprise visit to the Princess Alice Memorial Hospital at 9.30 A.M. and found everything in admirable order, the wards being specially well ventilated."

### PRESENTATION BY THE PRINCE OF WALES.

THE Prince of Wales last week opened the new hall and ambulance rooms of the Order of the Hospital of St. John of Jerusalem, at St. John's Gate, and subsequently presented the medals and awards voted by the chapter during the past year for deeds of gallantry in saving life on land at imminent personal risk. The first of these recipients was Miss Edith Lindsay, of Queen's Hospital, Birmingham, who was accompanied on the occasion by Miss Elkington, lady superintendent of the hospital. Miss Lindsay, it will be remembered, performed an act of great bravery at Bromsgrove last month, and endeavoured to save the life of a patient in her charge who suddenly rushed on the railway line in front of a train and was killed. She herself narrowly escaped a similar fate, being knocked down by the engine and badly injured. Happily, she has since recovered, and has had the gratification of having her courageous action recognised by the Grand Prior of the Order of St. John of Jerusalem. This is the first time that the silver medal has been received by a woman.

### APPOINTMENTS TO THE MILITARY NURSING SERVICE.

SOME further provisional appointments have been made to Queen Alexandra's Imperial Military Nursing Service. Miss J. G. Powell, Miss Young, and Miss H. Smart are gazetted sisters, and Miss A. C. Jacob, Miss L. F. A. Waller, and Miss K. Pearse staff nurses. It is also officially announced that the date of the provisional appointment of Sister J. E. Dods is June 17, 1903.

### THE NIGHTINGALE FUND.

THE report of the Nightingale Fund for the year 1902 shows that the number of probationer nurses who completed their year's training was 47, and the number remaining in the home at the end of the year 50. The number admitted in the period was 81, of whom 34 were discharged as unsuitable or left from other causes, 14 in their first month. The 47 who completed their year of training were placed on the register of Nightingale nurses and were taken on to the extra staff of St. Thomas's Hospital. The annual gratuity of £2 allowed by the regulations for the term of three years to the nurses who have completed a year's service satisfactorily was awarded to 96 nurses. Eight of the St. Thomas's nurses received the appointment of matron last year, one that of assistant matron, two that of night superintendent, and 11 that of sister. The death of Miss L. M. Gordon, the late matron, is referred to with regret, and the appointment of Miss H. E. G. Hamilton to St. Thomas's in her place is mentioned. The report embodies, as usual, the experience of the matron of the St. Marylebone Infirmary at her training school. She received 36 new probationers in 1902, while 21 completed their year of training and were taken on the staff of the infirmary, 10 leaving as unsuitable. Of former probationers trained in the school who had completed their engagement to the guardians, 17 received important appointments, seven becoming sisters at St. Marylebone Infirmary and two matrons of well-known institutions. Respecting the financial position of the Nightingale Fund, the payments by special probationers amounted to £620. The balance of income against expenditure was £159 8s. 5d., the expenditure including £244 gratuities to certified nurses and £150 grant to the Metropolitan Nursing Association for District Training.

### AN ORGANISATION OF RELIGIOUS TRAINED NURSES.

THE interest in the question of nursing by nuns continues. We are glad to be able to give some details of the Catholic Institute of Trained Nurses, which has been in existence for nine years, with the Dowager-Duchess of Newcastle as president, Lady Mary Howard and the Hon. Mrs. Fane as vice-presidents, Canon Murnane as treasurer, and a representative committee, largely composed of ladies, but including Canon Keatinge, two other priests, and two laymen. Last year, with the sanction and full approval of the Roman Catholic Bishop of Southwark, a step which had long been in contemplation was decided upon, and the nurses of the Institute have now joined themselves more closely together by a simple rule of life, in the hope of ultimately forming themselves into a Congregation of religious nurses. It is not proposed to adopt a religious dress, nor to make any changes which would be an obstacle to the sisters carrying on their nursing work with perfect freedom. The hon. superintendent, Miss Burd, informs us that in future no salaries will be paid, but that along with the simple rule of life it is intended to combine the best kind of nursing. None but nurses holding a certificate of three years' training from a general hospital will be admitted to the community. At present there are only three of these nurses who are working, as the nurses attached to the Institute have hitherto done, among the sick poor in the Roman Catholic parishes of St. George's, the Borough, and Bermondsey; but Miss Burd confirms the statement that the ambition of the organisation is to undertake private nursing also. Given full training and readiness to discharge all the usual duties of nurses, we can only wish God-speed to a band of women who occupy themselves with the nursing of the sick from the highest of all motives.

### A BRANCH OF THE JUBILEE INSTITUTE FOR SHEFFIELD.

A BRANCH of Queen Victoria's Jubilee Institute for Nurses has at length been started at Sheffield. The inaugural meeting at the Town Hall was of a representative character, and speeches in support of the movement were delivered by Alderman Franklin, the Bishop of Sheffield, the Lord Mayor, the Duke of Norfolk, the Master Cutler, and Sir Howard Vincent, M.P. An account of the inception of the Institute, its methods and work, was given by Miss Amy Hughes. As to the needs of Sheffield there is no room for doubt. Not only has it been up to now almost the solitary big town in the country without a branch of the Institute, but the nurses were only 20 in number, or less than 1 in every 2,000 of the population. Alderman Franklin was able to intimate the readiness of the Sheffield Board of Guardians to take up the scheme and to subscribe £200 a year towards the fund if at least four nurses were provided in the area of the Union. It has been decided to begin with six nurses, at an estimated cost of £100 a year each; and a very influential committee, with the Countess Fitzwilliam as president, has been formed.

### THE DUTIES OF A QUEEN'S NURSE.

THERE are curious conceptions still prevalent of the duties of a Queen's nurse. In connection with the new appointments which we published last week, the leading Welsh daily paper mentions that the names of no fewer than ten young ladies from Wales appear in the list. Our contemporary thinks it is quite possible that, in approving their appointment, the Queen bore in mind the time when the King, as Prince of Wales, passed through his serious illness many years ago; and adds, "When illness visits any member of the Royal house it is highly probable that the services of one or more of the ten Welsh nurses now on the staff of Queen Victoria's Jubilee Institute will be requisitioned."

### PROMISCUOUS BEGGING.

LAST week two nurses in uniform were seen on the esplanade of a fashionable watering-place in the Isle of Wight with a collecting-box, upon which was a placard stating that the contents were to be devoted to the Isle of Wight Infirmary and fund for district nursing. A local resident, knowing that neither of them was a district nurse, asked one if she came from the infirmary. She replied in the negative, volunteering the assertion that "she belonged to the London Hospital," but was "down for a holiday and had been asked to collect by a gentleman." She appeared to be a married woman, as she wore a wedding ring and a keeper, and went on to say that she knew nothing of the district. The resident suggested that if she was away for a holiday it would be far better that she should be resting and enjoying herself instead of running about with collection boxes. But this view was at once resented by the nurse, who maintained that she was enjoying herself vastly, and shook her box to show what a lot of money she had collected. Assuming that these nurses were actuated by the kindliest of motives, we agree with the resident that they were not turning their holiday to the best account. In fact, the practice of persons attired in nurses' uniform soliciting alms in public is not one to be encouraged, if only because it suggests to dishonest individuals the adoption of a becoming costume for purposes of their own. It is all very well and proper for nurses to assist at a bazaar or entertainment in the interests of the institution to which they are attached; but promiscuous begging on behalf of a charity with which they are not connected is under any circumstances a mistake, and it is a pity that a thoughtless public should encourage it.

### THE IMPERIAL SERVICE MEDAL.

IN the long list of recipients of the Imperial Service Medal there was the name of one nurse, namely, Mrs. Margaret O'Regan. Until she was pensioned, Mrs. O'Regan served at Kilmainham Hospital, Dublin, and at the time of her superannuation in October, 1902, she was senior nurse.

### RULES FOR WORKHOUSE NURSES.

AT the last meeting of the Gloucester Board of Guardians a letter was read from the Local Government Board with reference to the proposed revised rules for the workhouse nurses which have been submitted to them by the guardians. The communication of the Local Government Board was to the effect that they did not think it would be advisable to depart from their practice and express any opinion. The matter, it was added, was one for the guardians to decide. In the discussion that followed it was stated that some weeks ago, when a definite rule was submitted to the Local Government Board, they advised the guardians that they were acting *ultra vires*. Mrs. Hartland urged that the attitude now taken by the Local Government Board was perplexing, as in one case they said that the guardians must not make a rule, and in another that the Board would assume no responsibility concerning new rules. The guardians finally decided to put the revised rules into operation, a course which appears to be justified by the circumstances.

### FIRST AID BY A NURSE.

THANKS mainly to the pluck and presence of mind of a nurse, the life of a woman at Darwen was saved on Saturday. The woman's dress caught fire from a gas jet when she was cleaning the kitchen windows, and she was soon a mass of flames. Miss Polsne, a nurse, and the manager of a laundry opposite went to her aid, and her assistance was so effective that though the unfortunate woman was burnt about the lower parts of her body, she was later in the day pronounced to be out of danger.

### HOLIDAYS AND TEMPORARY HELP.

It is very desirable that nurses should have adequate holidays, but it is quite as important that in their absence from institutions the interests of the patients should not in any way be, or seem to be, neglected. The nursing staff at the infirmary under the jurisdiction of the Mutford and Lothingland Board of Guardians in Yorkshire will be one short of its number for twelve weeks during holiday time. At the last meeting of the Board the chairman proposed that the gap should be filled by the appointment of "a good useful woman who has been accustomed to a little nursing." This proposition, he said, had the approval of the superintendent nurse, and though a remonstrance was offered, it was not pressed. It does not appear to have struck the guardians that the argument in favour of choosing "a sort of motherly woman who was able to see after old women" is two-edged. Thus, it might be urged that, if for nearly a quarter of a year the patients in the infirmary can dispense with the services of one of the staff, it could as well be at once reduced. But the matter should be regarded in a higher light than this. Temporary nursing help should, of course, be of the same quality as that permanently given.

### A GIFT TO ROCHESTER HOSPITAL.

The trustees of St. Bartholomew's Hospital, Rochester, have been presented by Mr. Thomas Hellyar Foord, of Acorn House, Rochester, with a sum of £5,500 for the purpose of defraying the cost of building and furnishing a nurses' home. The gift is intended to perpetuate the memory of his parents and deceased brothers and sisters, members of the Foord family having previously been generous supporters of the hospital.

### NURSING AND "SHOP" COLLECTIONS.

The financial condition of the Blackburn District Nursing Association has improved, for the hon. treasurer was able at the annual meeting last week to state that the debt which was hanging over them last year had been removed by the receipts from the bazaar in aid of the organisation. But as the expenditure during the year exceeded the receipts by £245, there is still room for improvement. To promote that end, it is proposed that there should be a "shop" collection every year on the same lines as that taken for the infirmary. The idea is an excellent one, and might be adopted in other places, though "shop" collections should be supplementary to, and not instead of, regular contributions.

### A TRIO OF "NURSES" AT WIRRAL INFIRMARY.

At the last meeting of the Wirral Board of Guardians, the report by the medical officer in reference to the efficiency of the nursing staff was read, and Dr. Yeoman himself commented upon it. In his report he stated that the duties would compare favourably with any other place, that the nurses were not overworked, as acute cases never occurred more than one at a time, and that no increase in the nursing was required at present. But in his speech he said that when some changes were made in the nursing staff, he had asked to be supplied with one trained nurse and two probationers, and in place of one trained nurse, the guardians gave him one imperfectly-trained nurse, one with no training at all, and "one spoilt with a certain amount of training." At this stage one of the guardians not unnaturally remarked that he concluded Dr. Yeoman was, in spite of his report, dissatisfied, but the medical officer replied in the negative, and added that he was quite able to work with the staff he had got, which had been under his close observation for three months. A lady guardian had the courage to assert that, in spite of the satisfaction of the medical officer, a night nurse was needed in the infirmary where so many old people were near death, but her colleagues endorsed his report.

### A FUNCTION AT MARPLE BRIDGE.

An "At Home" for the benefit of the Marple Bridge Sick Nursing Association was given the other day at Erncroft, Marple Bridge. The proceedings included an address by Miss Amy Hughes, who dwelt at some length on two rules which, she said, had never been altered since the Queen's Jubilee Institute was founded. First, a Queen's nurse was not allowed to become an almoner ; and secondly she was not permitted to interfere in any way with the religious opinions of those among whom she worked. After tea, maypole dancing took place in the grounds to music, and in consequence of the long previous training of the 24 children, the figures round the maypole and on the lawn were effectively and gracefully carried out. The entertainment was repeated on another day, a small charge being made for entrance and tea, about 500 people paying for admission. The receipts amounted to nearly £10.

### SHORT ITEMS.

The nursing staff of Camberwell Infirmary have presented the infant son of the medical superintendent with a silver bowl and spoon.—An "At Home" was held last Saturday at the Ancoats Hospital, when the workpeople's committee and other friends were received. The latter included the Lord and Lady Mayoress of Manchester, who were escorted round the wards by the chairman, the matron, and the secretary.—Miss A. L. S. Dampier-Child, who was trained at West Ham Hospital, was married last month to Mr. Bertram Lovell, the friends present at the wedding ceremony in All Saints', Norfolk Square, Paddington, including the matron and night superintendent of the hospital, with other members of the nursing profession.—Through the kindness of Mrs. Brewis, wife of the chairman of the Portsmouth Board of Guardians, part of the staff of the Parish Infirmary recently enjoyed a day in the country at Waterlooville, a charming Hampshire village, the programme including tennis, a strawberry tea, and a ramble in the woods.—Miss S. R. Alice Rimmington, who is retiring from the post of lady superintendent of Bancroft's School, Woodford Green, has taken a high-class boarding-house in Regency Square, Brighton.

### The Nursing Outlook.

" From magnanimity, all fear above;
From nobler recompense, above applause,
Which owes to man's short outlook all its charm."

### THE RESPONSIBILITY OF TRAINING SCHOOLS.

Last week we considered the so-called methods of training which do not involve three years in hospital and adequate teaching as well as experience; and we suggested that the smaller and more special fields might be of use if the bigger training schools would co-operate and act together with them. Some of the big hospitals are engaged in a feverish rush for funds, and have hitherto shown no desire to co-operate. Whenever competition is keen the care of the nurses may be forgotten in the fight to maintain a foremost position. Then, too, the nurses are generally quiet, unselfish women, who allow their needs to go to the wall, and so the committee and the officers do what they will. All the more reason why hospital officials who are really concerned for the welfare of the sick as a whole should carefully consider whether their nurses are efficiently trained in an all-round manner which will make them valuable servants of the community. This view is too often overlooked where a committee aims at training for their own wards only. In a few hospitals which do not need the nurse always in the wards they send her out and make what profit they can out of her earnings, but they leave her without any pension provision, or perhaps with a pension that depends on the will of the committee.

It is not enough to give a nurse three years' training and a certificate, and then think that the whole duty of the hospital towards that woman is done. That is a small and selfish view; and yet in the troublous days which are past gratitude to the hospitals that did even this much overcame the consideration of what more was necessary. For there can be no doubt that the proper term of training before which a certificate can be earned is three years, and that if a nurse wishes to specialise the special training should be laid upon the bed-rock of the three years of probation. If a man wishes to become an oculist he does not go first to an eye hospital for six months and then start as a practitioner. He takes his full five years of medical training and then he tries to get added experience in the special branch that attracts him. In the same way the nurse's three years in a general hospital should be the basis of all future experience. But at the end of the three years the nurse should not consider herself a "finished product." Every hospital has its own methods and mannerisms; every medical man and every matron have their own fads and fancies, and the treatment of to-day is often the quackery of to-morrow. And so if a nurse is going forth from her big training school to other branches of work where she will rub against all sorts and conditions of medical men, or perhaps in some colonial station be obliged to assume responsibility of her own, it is most necessary that her training should not have been too mechanical, too much the daily routine of a well-appointed ward with every convenience at hand. Here is where co-operation might come in: the Seamen's Hospital has made arrangements by which its nurses spend six months in a woman's hospital, and we can imagine no better experience for the nurse who has been two years in a big London hospital, and who the sister sees is in danger of becoming a mere machine, than to be sent for six months to some cottage hospital where it may once more dawn upon her that a patient is a human being. For we confess that we are looking on the nursing profession as a whole—not considering only the gracious and capable hospital sister who is so perfectly adapted to her post in an English ward. We are thinking of the patients—rich and poor—in their own homes; of the wretched pauper whose pitiful poverty is being ended by death in some Workhouse infirmary, of our sons who have taken up the white man's burden in far-off lands, of our dusky subjects in India dying of famine and the plague. To fit a nurse for hospital duty her training must not be narrow—her school authorities must look beyond their own walls.

There is a London Central Council of the twelve big hospitals of London whose duty and function it should be, if it is ever to fulfil any useful duties or functions, to consider what action should be taken to improve the *whole status* of the nursing profession and to set a standard for the smaller training schools which will serve them for an example. It is the duty of this Council to meet and thresh out the whole subject of the present unrest and lack of co-ordination in nursing circles, and by their own co-operation and courtesy to aid in the co-operation of all nurse-training schools, big and little, towards the evolution of the perfect nurse. In this way only can the half-equipped and wholly faulty nurses be driven out of the field, and be replaced by capable women of character, skilled yet kindly, learned yet modest. And it must be an understood principle that each nurse receives her own earnings, and that her future is provided for by an adequate payment to the Royal National Pension Fund. There must be no more sweating of the trained nurse for the good of the hospital, for the true worker is worthy ot her hire; the properly trained nurse is capable of controlling herself; and the profession is putting ott its baby clothes.

The public and the nurses properly look to the great nurse training schools to live up to their responsibilities, in this matter, at the present time. It will be properly put down as marking their failure to discharge an important trust should they longer remain inactive; divided amongst themselves; indifferent when it is shown that they have fostered by neglect the perpetuation of the wooden machine or half-trained nurse, in lieu of the perfect nurse who alone should possess the three years' certificate of training. An adequate minimum standard of nursing knowledge and efficiency is imperatively called for. We look to the nurse training schools in co-operation to evolve such a minimum standard without delay or further hesitation.

### Lectures on Medical and Surgical Nursing.

By John Hopkins, F.R.C.S., Medical Superintendent of the Central London Sick Asylum District Asylums, Cleveland Street, W., and Hendon, N.W.

### LECTURE XII.—DISEASES OF THE DIGESTIVE SYSTEM.

The mouth and pharynx, through which food and air enter the body are peculiarly exposed to the causes of inflammation, as bacteria and their spores are brought by this means into contact with the mucous lining of these parts. The inflammation may be accompanied by ulceration, phagedæna and even gangrene. Tonsillitis is a common complaint. When acute and leading to abscess it is known as *quinsy*. When chronic the tonsils enlarge and cause obstruction to the free entrance of air into the lungs, and in children in whom the ribs are soft and yielding, the efforts to inspire cause the chest walls to fall in, and thus there is a deformity produced. Adenoids are enlargements similar to the tonsils that lie above the soft palate and obstruct the breathing through the nose. Children troubled with them breathe with the mouth habitually open, and this gives them a characteristic facial expression.

*Thrush* is due to the growth of a fungus on the mucous membrane and has the appearance of white patches. It is seen in children and is often accompanied with diarrhœa. The same thing is seen in phthisical patients when the disease is advanced. The use of mouth-washes, gargles, sprays, douches or swabs is required in diseases of the mouth and throat, and they need to be thoroughly and systematically applied.

*Stricture of the Œsophagus* causes difficulty in taking food, and may be simple or malignant. Solids when swallowed tend to lodge at the seat of obstruction, and after a while they are regurgitated in an unaltered condition. Liquid food must then be relied upon, but this also may be unable to find its way into the stomach. The stricture is sometimes prevented from closing completely by a tube that is passed through it and constantly worn. When it is impermeable, as a last resort to prevent the patient from dying of starvation, an opening can be made into the stomach through the abdominal wall. This operation is called *gastrostomy*.

The stomach, if improper food be taken, will empty itself by vomiting. Irritant poisons will cause vomiting and at the same time set up inflammation or gastritis. Corrosive poisons will destroy the mucous lining, which may be vomited whole. Gastritis may be present in various acute diseases, such as fevers, pneumonia, and acute bronchitis. It is also seen in disease of the heart, and of the kidney and in gout. The feeding of the patient is of importance in all such cases. In children the milk often causes trouble, and special care is required in preparing it and in attending to the bottle. Indigestion is a common result of constipation. It is quite notable how little indigestion is complained of by the inmates of an infirmary, where attention is paid to the action of the bowels.

The *appetite* is a matter deserving of attention. *Anorexia* or loss of appetite is of common occurrence. *Bulimia* or excessive appetite is a symptom of diabetes, and in certain diseases of the brain patients eat voraciously. *Pica* is the name given to a perverted appetite in which substances are greedily partaken of that are not food.

*Vomiting* is a frequent event and occurs in a variety of bodily states. Observation as to the nature and quantity of the vomited matter, and as to the time and manner of vomiting is important.

*Hæmatemesis* or vomiting of blood is most often due to ulcer of the stomach and congestion of the liver. When in small quantity it becomes altered in appearance, and may look like coffee grounds. It is sometimes a question as to whether the blood has come from the lungs or the stomach, so it is important that the vomited matter be saved for inspection. At the same time a nurse should be able to judge as to the probable source from the colour of it and the presence or absence of the contents of the bronchi or stomach.

*Melæna* or passage of blood from the bowel may occur when bleeding is taking place from the stomach or duodenum, in which case the dejecta are black and look like tar. Blackish fæces are caused by certain medicines—iron and bismuth. When blood comes from the rectum it may be in streaks or small clots, or it may be very abundant.

*Gastric Ulcer* is a dangerous disease, because the floor of the ulcer may be perforated and let the food escape into the peritoneal cavity, and thus set up fatal peritonitis. The feeding is of the greatest importance; anything indigestible or a large meal might cause the ulcer to give way. An aperient might be sufficient to bring this about.

*Cancer* may attack any part of the stomach. When it invades the pylorus the food is prevented from passing through it and the stomach dilates. The vomited matter is in large quantity, containing perhaps several meals. Temporary relief may be obtained by short circuiting the stomach with the bowel by a surgical operation.

*Colic* is a painful spasm in the muscular coat of the intestine. The patient may be doubled up with the pain. Similar spasm taking place in the bile duct, ureter or uterus is also called colic. True colic of the bowel is generally due to constipation. Lead taken into the system will cause colic among other symptoms, and the pain may be excessive.

*Constipation* is the source of a variety of troubles such as indigestion, want of energy, depression of spirits and anæmia. The bowels may act every day and yet there may be a steadily increasing constipation until the colon is loaded. When the rectum is loaded there is a constant desire for the bowels to act, and sooner or later the irritation caused by this condition causes a catarrh and there is frequent evacuation of liquid fæces. Injections may relieve this, but often it becomes necessary to resort to mechanical means. Constipation tends to increase with the use of aperients, so their habitual use is to be discouraged, and regular habits inculcated.

*Diarrhœa* is generally caused in healthy people by some irritant, often fermenting food. In summer especially is diarrhœa dangerous in children. This renders it very important that care should be taken in selecting and preparing their food.

*Chronic diarrhœa* is caused by tubercular ulcer, lardaceous disease or malignant growth, but it sometimes occurs and persists for months without any such cause.

Rest and warmth are essential and the feeding has to be carefully regulated. Raw meat finely grated into a pulp may be the only food allowed, especially for infants.

*Appendicitis.*—The appendix to the cæcum is especially liable to inflammation. Its mouth may become blocked and then suppuration in the blind sack beyond causes ulceration and perforation into the peritoneal cavity. This would in all cases prove fatal but for the gluing together of the intestines round about the appendix by inflammatory lymph before the actual perforation takes place. Even then the pus may increase and the abscess thus formed burst into the peritoneal cavity and so set up general peritonitis. Sometimes an abscess forms behind the cæcum, and causes the same symptoms as appendicitis. No aperient should be given in these cases nor any solid food, and absolute rest is imperative. An operation is required to let out the pus or to remove the offending appendix.

*Colitis* gives rise to the passage of abundant clear mucous and if there be ulceration this may be mixed with blood. A membranous cast of the bowel may be passed or bits of mucous membrane.

*Hæmorrhoids* or piles may be external or internal. They are due to varicose veins, which are distended and dilated by straining.

At first, only evident during the act of straining, they tend to become permanent and may bleed freely, or become inflamed and painful. The blood may clot in them and they then become solid. They are treated by ointment, rectal injection and by regulation of the bowels and of the general health.

When inflamed there is much pain and soothing applications are needed. Operations for the removal of piles are by ligature, clamp and cautery, and excision.

*Fissure of the Anus* is an exceedingly painful crack in the mucous membrane generally situated posteriorly. The distress after action of the bowels is very acute and digital examination is impossible. A quick and effectual remedy is a simple operation under an anæsthetic.

*Fistula in Ano* begins as an abscess by the side of the rectum in the ischio-rectal fossa. This bursts either internally or externally, and the sinus remaining cannot heal on account of the action of the sphincter ani. By cutting through this under an anæsthetic the parts are set at rest and the fistula heals. The incision should be dressed by packing in order to insure its healing by granulation from the bottom.

*Cancer of the Rectum* causes a discharge of mucus, blood, and pus, and there is a strong fætor. If situated within reach the cancer may be removed by operation, together with the lower part of the bowel.

In *Preparing Patients for Operation* about the rectum the object must be to have the bowel where possible well cleared by aperients and injections before the patient is placed upon the operating table, and care is necessary to avoid a loose action of the bowels during the operation as a result of the previous attempt to clear them.

### Nursing in the Mission Field.

BY A CORRESPONDENT.

NURSING is one of the most interesting branches of the work in the mission field, and one by means of which we can more easily win the affection of the native people. It was amongst the Kaffirs I was working, and the tribes I came in touch with were the Tambookies, Basuto, Fingoes, Zulus, and Hottentots. The Tambookies were a very warlike, fine-looking race, and were very seldom to be counted among our patients, while the Fingoes were often on the "sick list." It is when they become Christians and wear European apparel that they feel the cold so much, because it is when they change their mode of living and leave off their skins and blankets that they leave off also the grease with which they rub themselves all over to fill up the pores of the skin and keep out the cold. The natives on the whole make very good patients, especially the surgical ones. They do not seem to mind the extent of any outward wound or accident, so long as they can see it with their eyes, but it is when they are inwardly ill that they are very frightened, and their one cry is, "Si ya fa, si ya fa!" that is, "We shall die, we shall die!" One year influenza was very rife amongst the natives, and three or four of our native girls—we had a native girls' boarding school—had it. They were too miserable to do anything but lie rolled up in their blankets on their mats in their bedroom. They told me they could not see they were ill, and therefore it made them feel much worse.

### THE FADS OF NATIVES.

The natives believed thoroughly in our medicines so long as we coloured them with either pot. permang. or cochineal; but if we gave them in their natural colour, *i.e.*, white, however nasty the taste might be, they would not beieve in them. For colds in the head they had their own treatment. The wormwood, which grows in great quantities as a weed, has a very pretty feathery foliage, which has a very strong odour. They pick these leaves and push them up their nostrils, when they are supposed to take away the cold. With regard to surgical cases, they heal up very quickly as a rule, owing, I think, to the beautifully clear atmosphere. One of our small schoolboys cut his forehead open rather badly with an iron hoop just as he was going into school. We took him in, washed the wound, which was very deep, and drew it together with stamp paper! (we possessed no strapping at that time) and sent him home to lie quiet in his hut for a few days. This he did, at the end of which time he returned with the wound healed beautifully and hardly a scar to be seen.

### A TEST OF ILLNESS.

Two accidents were reported to us but I cannot vouch for the truth of them, as I did not see either of them. One was that a native had thrown an assegai at a companion and it had gone through the man's chest and out between his shoulders, but that he was doing well, having lain quiet in his tent for a few days. The other was that a native had fallen near a passing waggon and one of the wheels had gone over his head! No bones were broken—the natives' heads are very hard—and he was better after a few days' rest in his hut. It is very funny and very difficult to find out what is the matter with native patients when you first see them. They may perhaps tell you, "I am with a head," *i.e.*, "I have a headache," but this is as much as they can explain. Then until you are used to their dark complexion you cannot possibly tell by their looks, but after a time if they are really ill their pretty bronze complexions become a dull yellow colour and the whites of their eyes also change colour.

### AN ADVENTURE WITH A SNAKE.

While I was out on the mission field a very amusing incident occurred, which turned out to be a "novel cure for hysteria." A colonial woman who had been very ill in our house was nearly convalescent, but did not like the idea of helping herself in the smallest way. She pretended she could not possibly sit up in bed, nor feed herself—in fact, exert herself at all. One lovely typical African summer-day she was carried out into the garden to lie amongst the trailing vines under the willow trees. She had been lying there, immovable, for some time, when suddenly I heard a cry, "Oh, Sister come quick, a snake, a snake!" I went out on to the stoep, called the sister who was looking after her, and told her what was happening. She ran into the garden and found the patient had moved quite away from where she had put her, but that the snake, a cobra, had followed her and was sitting spitting at her. As soon as the snake saw the new arrival it made away, and was never seen again, nor had one been seen previous to this, but it had done its duty, and we were all very grateful to it for its visit that day. As a result the patient never gave any more trouble, the hysteria disappeared, she helped herself all she could, and soon got quite well again.

The Need of Knowledge.

I strongly advise nurses who hope to go out to the mission field to learn all they can with regard not only to the nursing but also to the treatment of the patients in cases of emergency, for sometimes we are stationed several days' journey from the nearest doctor. People have often said to me, "Oh! how can you like nursing the natives, I could not possibly do such a thing?" They have never had any opportunity of doing so, but I can only say it is a deeply interesting work and one which helps very much in winning the natives, for at first they look on you as it were at a distance, but soon when they see you really care for them and take an interest in them, they soften down and soon trust you.

### Everybody's Opinion.

[Correspondence on all subjects is invited, but we cannot in any way be responsible for the opinions expressed by our correspondents. No communication can be entertained if the name and address of the correspondent are not given as a guarantee of good faith, but not necessarily for publication. All correspondents should write on one side of the paper only.]

### THE LATE MR. W. E. HENLEY.

"A Reader" writes: Now that the time is fast coming when every infirmary in the United Kingdom will have its thoroughly-trained and efficient nurses to wait upon the sick, it is interesting to note the condition in which Robert Louis Stevenson found his friend, W. E. Henley, when he was taken in 1878 to see him in the Edinburgh Infirmary. He says: "The poor fellow sat up in his bed with his hair and beard all tangled, and talked as cheerfully as if he had been in a king's palace." Evidently, hair combing or beard brushing was not part of the nurse's daily duty in those days, though one must not forget that the "nursing," such as it was, under Dr. Lister's care saved the crippled poet's remaining leg.

### SHORT-TRAINED NURSES.

"Justice for All" writes: I wish to air a grievance in your paper which a great many nurses consider ought to be remedied. It is in connection with the short-trained nurse. Ten years ago a year was considered by many of our largest hospitals sufficient training for private nurses, consequently many were trained for that length of time. These nurses now find it most difficult to obtain good posts. Though they may be in every other way suitable, they are placed on a level with any young nurse who may have been in hospital for a year. In most professions or trades when improvements are made, the case of old workers is considered. I consider that there ought to be some sort of examination which a nurse trained previously to a certain date might pass and thus become fully qualified.

### THE BEST WAY TO TREAT MOSQUITO BITES.

"Sister" writes: In July, 1901, I was nursing at a country house where there was a pond near the garden, and we had quite a plague of mosquitos. I was each day badly poisoned by them, my head, neck, arms, and hands being quite disfigured. I found the mosquitos were greatly attracted by *blue*. I always got more bites when in my blue cotton dresses than when in other colours, and I was afterwards told that blue attracts the mosquito in the way red attracts the bull. The use of bathing with ammonia never relieved me, and my friends—other victims—told me the same thing. I found the following the best lotion to use, and as it was my own idea I feel I may safely pass it on to others. Mix with half pint 1 × 20 carbolic 4 ounces 4711 Eau de Cologne. After the morning bath, bathe the face, neck, hands, and arms with Eau de Cologne and water. This, to a certain extent, keeps away many mosquitos. When stung by them, at once, and frequently during the day, dab the places with the preparation. This takes down the swelling, and allays the irritation. I have given this advice to some golf players, and I hear they find the lotion of great service to them.

### STATE REGISTRATION.

"H. T." writes: Many nurses will be grateful to Princess Christian for her kindly and sympathetic remarks at the annual meeting of the Royal British Nurses' Association over the discussion of State registration for nurses. The Princess said "that there were many good nurses who had not had a long training, and they must be fair to them." If State registration, as apparently regarded by the Association, were to become compulsory, would it not mean that all who hold a certificate for less than three years would not be eligible for the roll, and therefore would not be entitled to be considered "trained" in any sense? I think that if those who are so eager for this to be hurried on would take into consideration the unfortunate women who were trained twelve or fourteen years ago, when one year's residence in a large general hospital or infirmary was the rule for the majority of nurses, they might look at the question in a different light. Many of these nurses are now beyond the age limit for further training, or for other reasons cannot begin again. Are all their years of experience and constant practice to count as nothing; are they to be branded as incapable of attending to the sick, and their livelihood practically taken from them? A long course of hospital training is most desirable, but in common justice years of experience in nursing should be recognised as well.

### "THE MATRONS' COUNCIL" AND STATE REGISTRATION OF NURSES.

"A Hospital Sister" writes: As a nurse, may I say a few words as to the pretensions of the above "Council"? What shadow of title has this self-elected, non-representative body to the position it arrogates to itself as leader of a mission "to bring about a uniform system of education, examination, certification, and State registration for nurses in British hospitals"? Imagine a self-elected body of men calling itself "The Council of Public School Headmasters" on whose list of "councillors" or "members" the names of the headmasters of Eton, Harrow, Charterhouse, Westminster, Winchester, etc., were, with one exception, conspicuous by their absence, and in their place the names of the headmasters of provincial grammar schools, and you have a parallel to "The Matrons' Council." Could such a body be considered in any way to represent the headmasters or the public schools? and would not its members be very much laughed at if they aspired, as "*The* Headmasters' Council," to control the public-school education of England? Such an important matter as the State regulation and control of the nursing profession must of necessity be taken up and organised by the leaders of the nursing profession, *i.e.*, the heads and representatives of the great London and provincial training schools, and not by an insignificant minority. It is not a question of "the whole nursing world," which apparently the "Matrons' Council" imagines itself to represent (I believe the "Society for the State Registration of Nurses" has about 800 members), "standing still because four of its members have not realised the value of co-operation." It is a question of the real nursing world, as represented by the great hospitals of England, waiting to advocate and press for a measure which will change the whole future of the nursing profession until the need for such a change and its wisdom are considered by them to be sufficiently proved to justify their taking action in the matter. Then there is little doubt they would succeed in getting a Bill passed providing for such legislation. Until then, an agitation, engineered by irresponsible people, will rather retard than hasten nursing reform.

### WAGES FOR VILLAGE NURSES.

"S." writes: I was much interested to read the article in THE HOSPITAL *Nursing Section* on the "Living Wage of the District Nurse." So much is written now condemning the employers of "underpaid" village nurses, that these few lines may not seem out of place. I do not write to excuse underpaid labour, but I do think it is a pity that so much is said and written to discourage women from becoming village nurses by putting ideas of exalted grandeur in their minds. It is very difficult to know how best to help the suffering cottagers. Most of the readers of THE HOSPITAL should know the difference between the "district" and the "cottage" nurse, though from personal experience I find that few matrons or nurses do know anything about the "cottage or village nurse," and some go as far as to say to me: "I know nothing about the cottage nurse except that the whole system is opposed to my ideas of nursing, and that it is quite contrary to my principles"! But what a narrow view to take. Why condemn a whole system, when a little careful thought will show that it has its good side too? No one could be more interested in nurses, their profession and their welfare than I am; no one would willingly be less anxious to offer them a "starvation salary," as a village nurse's salary has so often been called. But one has two points to consider; one wishes to do the best for the nurse and the best for the cottager. Doubtless, in a thickly-populated district, the service of a thoroughly-trained district nurse, who has the advantage of being a lady by birth, and who carries too a spiritual atmosphere with her wherever she goes is an ideal state of affairs. But in a thinly-populated village, where there are at most periods of the year but one or possibly two cases to attend to at the same time, what would be the good of an educated nurse who would only visit the patient for a short time and attend to her immediate wants and leave again. Instead of committing suicide because she "had no one to talk to," the nurse might be inclined to do so for "want of work." There is no one in the village who can be spared to look after the rest of the family or do the necessary housework; this is no reflection on the district nurse, it is not her work, nor is it required of her where there are plenty of inhabitants, and a "friendly neighbour" can usually be found, though I know that no real lady refuses any work that presents itself to her. But in a small village, where during such times as hop-picking every house is forsaken, when a confinement occurs, there is absolutely no one to call in to help the doctor or do the mother's work, as she has not always relations to summon, and the patient and infant—to say nothing of the other children and the house—are left quite uncared for. Cases such as this have come before my notice several times in my own village, and is not a cottage nurse then an immense boon? I require a nurse in my village; a "district nurse" would be useless because there are so few inhabitants, to join with any neighbouring village and employ a "district nurse" then, would be equally impossible on account of its position, and I want to do the best for the nurse and the cottagers. It is not a question of funds, as I feel sure I could collect any sum necessary with the kind aid of my friends, but if we paid an uneducated woman at the rate suggested in the article last week for the district nurse, would she not consider herself "too grand" to scrub the floors and cook the dinner for the family? I cannot see why the whole system of village nursing should be so condemned, and I trust I may have convinced a few patient readers that the village nurse is the right woman in the right place at times, as is the district nurse in hers, and let us rather encourage the "working class" of women to train for such a post, instead of frightening her with notions of her being "underpaid." I quite agree she should be fully trained as a midwife, too, if she is to attend confinement cases without a doctor, but I do think it is a wiser plan to give her the salary that befits her station in life, as by paying her too highly we only unfit her for a position for which the woman taken from the class she is to nurse is so admirably suited.

### Appointments.

[No charge is made for announcements under this head, and we are always glad to receive, and publish, appointments. The information to insure accuracy should be sent from the nurses themselves, and we cannot undertake to correct official announcements which may happen to be inaccurate. It is essential that in all cases the school of training should be given.]

HOSPITAL FOR WOMEN, SOHO SQUARE, LONDON.—Miss Mabel Hartley has been appointed staff nurse. She was trained at the Royal Infirmary, Halifax.

LANCHESTER WORKHOUSE INFIRMARY.—Miss Frances A. V. Young has been appointed superintendent nurse. She was trained at the Royal Infirmary, Newcastle-on-Tyne.

NORFOLK AND NORWICH HOSPITAL.—Miss Maud O'Rowden has been appointed home sister. She was trained at Adelaide Hospital, Dublin.

NORTH DEVON INFIRMARY, BARNSTAPLE.—Miss Helena Good has been appointed sister. She was trained at the Royal Albert Hospital, Devonport.

PORT OF LONDON SANITARY HOSPITAL, DENTON, NEAR GRAVESEND.—Miss Lizzie Muir has been appointed nurse-matron. She was trained at Lightburn Fever Hospital and the Small-pox Hospital, Berwick-on-Tweed. She has since been nurse-matron at Gastro Hospital, Skye, temporary charge nurse at Lightburn Fever Hospital, charge nurse at Edinburgh Poorhouse Hospital, and nurse-matron at Berwick-on-Tweed Small-pox Hospital.

ST. MARY ISLINGTON INFIRMARY.—Miss Annie Williams has been appointed sister. She was trained at the General Hospital, Birmingham, where she was afterwards staff nurse. She has also been charge nurse at the Brook Hospital, London.

SALOP INFIRMARY, SHREWSBURY.—Miss M. M. M. Brown has been appointed night superintendent. She was trained at Perth Royal Infirmary, where she was afterwards staff nurse. For nearly three years since she has been employed as nursing sister in South Africa, and at the Military Hospital, Colchester. She holds the L.O.S. and Glasgow Maternity Hospital certificates.

SHEFFIELD ROYAL HOSPITAL.—Miss Amy McLean has been appointed sister of the out-patient department. She was trained at the Liverpool Royal Infirmary and has since been sister of the Women's Medical Ward at the General Hospital, Wolverhampton.

STROUD HOSPITAL.—Miss Sarah Tyson has been appointed charge nurse. She was trained at Stroud Hospital.

UNION INFIRMARY, COWLEY ROAD, OXFORD.—Miss Julia McMahon has been appointed assistant nurse. She was trained by the Meath Workhouse Nursing Association at the Children's Hospital, Temple Street, Dublin.

VICTORIA HOSPITAL, FOLKESTONE.—Miss Kate Halsey has been appointed night sister. She was trained for three years at the North-Eastern Hospital for Children, Shoreditch, and for three years at King's College Hospital, London.

WALSINGHAM UNION WORKHOUSE INFIRMARY.—Miss Elizabeth Margaret Stone has been appointed superintendent nurse. She was trained at the Royal Infirmary, Newcastle-on-Tyne, and has since been charge nurse at Tynemouth Union.

WEST HAM UNION INFIRMARY.—Miss Mary Read has been appointed home sister. She was trained at St. Thomas's Hospital, London, and East London Hospital for Women and Children, Shadwell. She has since been sister at Monsall Fever Hospital, Manchester; sister at Bethnal Green Infirmary, and sister and temporary night superintendent at Islington Infirmary, Highgate.

RADCLIFFE, RAMSBOTTOM, WHITEFIELD, AND BURY ISOLATION HOSPITAL.—Miss Nellie Sinclair, the new charge nurse, was junior assistant nurse, not charge night nurse, at Enfield Cottage Hospital.

### Echoes from the Outside World.

### Movements of Royalty.

THE two days which the King spent at Eastbourne have given much pleasure to the inhabitants of that fashionable seaside resort. Although the visit was intended as a private one to the Duke and Duchess of Devonshire at Compton Place, his Majesty, learning that it was desired to give him a public welcome, gave his consent and promised to receive an address and to drive along the principal streets, which, in consequence, were beautifully decorated. The King, who arrived at the station on Saturday ten minutes before he was due, was escorted by four mounted constables, and one of the horses becoming restive backed into one of the wheelers of the Royal carriage. Fortunately, the carriage was at once stopped and no harm was done. On Sunday the King, accompanied by his host and hostess, walked to St. Peter's Church, a few hundred yards distant, and attended morning service, and in the afternoon he drove by motor car to Polegate to see the Duke of Devonshire's stud farm. As early as half-past nine on Monday morning the King drove to the Princess Alice Memorial Hospital and paid a surprise visit. Subsequently, on his way to the station, a well-known local character, Kitty, "the Royal flower-girl," threw a bunch of carnations into the King's carriage, which he acknowledged with a smile. On the occasion of a former visit to the town the King had accepted a flower from the girl, hence her name.

On Saturday afternoon the Queen with Princess Victoria drove down to the grounds of Hurlingham Club to witness the final of the inter-regimental polo tournament. The Prince and Princess of Wales were expected, but no notification of the Queen's intended visit had been received, and as she had not been to Hurlingham for several years, the gatekeeper did not know her, and required an explanation before he would allow the Royal carriage to pass. But the driver of a four-wheeled cab outside the grounds had recognised her Majesty at once. The carriage-way to the gates of the club grounds is rather narrow, and as the Queen drove up it, he was bidden by the policeman on duty to drive on. "No," was the reply, "I ain't a-going on in front of she."

### The Queen's Kindness.

AN old lady of nearly ninety-three years of age, Mrs. Esther Marler, of Taunton, who is still sufficiently active to be able to attend a Sunday school every week, has received the following letter from Queen Alexandra:—"Miss Knollys is commanded by the Queen to thank Mrs. Marler for the pretty tea cosy she has kindly sent, and to say that, although it is against her rule to accept presents from anyone with whom she has not the pleasure of a personal acquaintance, yet, in view of Mrs. Marler's advanced age and the kind spirit in which the gift was offered, her Majesty will make an exception in Mrs. Marler's favour and will be happy to keep the cosy. Miss Knollys is further commanded by the Queen to forward Mrs. Marler a Coronation cup and saucer, thinking she would like to have a little souvenir of that important event."

### Ball at Marlborough House.

THE first ball since the Prince and Princess of Wales entered into possession a year ago was given at Marlborough House on Monday evening. Over a thousand guests had been invited, and to accommodate them the ball-room, the supper-room and the adjoining apartments had all been erected in the garden. The decorations of these temporary structures were exactly similar to those on the walls of the rooms of the house itself, so that those who failed to look upwards at the canvas roof would have believed Marlborough House to be a much more capacious building than it really is. Ivy-leaf geraniums, with their pretty pink blossoms, and marguerites played an important part in the scheme of colour, and the whole was lighted by electricity. The same motive power was used to drive the fans and insure the coolness of the apartments. The King and Queen, the Prince and Princess of Wales, and other members of the Royal family watched the dancing from a raised daïs, and a smoking-room for the use of the Royal party had been erected in a corner of the garden in East India style. The dining-room of Marlborough House was used for the Royal supper-room.

### The Pope's Illness.

ALL hope of the recovery of the Pope is abandoned. The bulletin issued on Wednesday morning at 9 o'clock said: "There is no change in the patient's general condition." King Edward having addressed a letter to Sir Francis Bertie, our Ambassador, making inquiries regarding the condition of his Holiness, Monsignor Stonor goes twice a day to the Vatican and afterwards communicates the news received by him there to the British Embassy, taking with him the bulletins.

### The Welfare of the Feeble-minded.

THE annual meeting of the National Association for Promoting the Welfare of the Feeble-minded, by the kind invitation of Lady Frederick Brudenell-Bruce, was held at 11 Gloucester Terrace, Regent's Park, last week. Miss Louisa Twining moved the adoption of the seventh annual report, which showed that the work of the Association was progressing, and that the general outlook had brightened. Although little had been done by Parliament, the Poor-law authorities of London and the larger towns more especially were becoming increasingly alive to the necessity of dealing with defective children, though in rural districts they still remained without any special provision. A resolution was carried that the meeting was strongly of opinion that a Royal Commission should be appointed to investigate and report upon the condition and needs of feeble-minded persons throughout the country. One of the speakers emphasised the fact that they did not want to promote legislation till they had had an inquiry, and that what they wanted was a travelling commission to get information from all parts.

### Death of Mr. W. E. Henley.

BY the death of Mr. W. E. Henley at Woking last Saturday the world of letters and of English poetry lost a notable figure. Like his friend and collaborateur, Robert Louis Stevenson, he was a great invalid. When little more than a boy he was attacked by a disease which necessitated the amputation of one foot. Later he was told by the doctors that the sacrifice of the other leg was necessary if he desired to live. But he had heard of the fame of Dr. (now Lord) Lister, and determined to see whether something could not be done to prevent the loss of a second limb. Penniless, and almost friendless, he travelled third-class to Edinburgh, though suffering much, and with only a few shillings in his pocket reached the infirmary. Here he was admitted, and his faith in the great surgeon justified, for the leg was saved, though he had to remain a patient for nearly two years. As a result he wrote his first book of poems, "In Hospital," and it was in the infirmary that he made friends with R. L. Stevenson, who was brought by an acquaintance to be introduced to the crippled poet. In 1877 he became editor of a weekly paper, and in 1878 he married the lady whose affectionate and heroic sympathy did so much to cheer and sustain him all through his life of constant ill-health. Subsequently he was in charge of "The Magazine of Art," but it was "A Book of Verses," published in 1888, which first caused his name to be widely known.

### Two New Books.

### I.—STUDIES IN THE WORKS OF THREE ENGLISH-WOMEN.*

MANY criticisms of the writings of the three great women novelists, Charlotte Brontë, George Eliot, and Jane Austen, have been attempted from time to time, but none more able, nor unbiassed than the "Studies" of Mr. Bonnell have appeared of recent years. Sympathy and insight are conspicuous throughout. Mr. Bonnell quotes at starting the biographer of Rénan. Three principal influences go to shape human character; that of heredity, that of locality, and that of everyday association. But "any approach to finality in criticism may be despaired of until the image shall have passed. . . . into an atmosphere which has ceased to pulsate with the passions and prejudices, the friendships and hatreds of the present hour."

Charlotte Brontë loved Nature as all her readers know; it was to her no sealed book, but a solemn mystic consoler to whom she turned in that daily life so bravely borne by the gifted sisters, in their isolated parsonage on the edge of the bleak, Yorkshire moor. Mr. Bonnell considers that there is no painter of scenery, no painter of atmosphere like her. As to the genius of Charlotte and Emily Brontë in comparison to that of George Eliot, he says that the pure genius of the former was "unsullied by the alloy of learning," and "Because of her narrower horizon, Charlotte Brontë had a more compelling genius than George Eliot, whose acquaintance with the world's philosophies so overlay her thought that the piled up learning was constantly threatening a blockade of the tap root of genius, whence flow the living juices which colour the whole. . . . Innocence of the life of the world energises and drives down to its deepest springs the search into the life of self. The utter absence of world-knowledge becomes the utter presence of self-penetration. . . . George Eliot's genius shone through talent, Charlotte Brontë's in spite of talent. Only the errors of genius led astray are more far-reaching than the accidental lapses of genius pure and simple."

In summing up his impressions of Charlotte Brontë, Mr. Bonnell writes:—

"As for me, whenever a purple sunset streaks the West, whenever the moon rises 'in an element deep and splendid,' whenever the soul swells with killing pains on silent moon-filled nights, I think of Charlotte Brontë—I think of the pang of all the world, the unutterable cries . . . which well up into the pitying skies and shine there on the stricken earth. Whenever I lie awake in the night-watches listening to the wind . . . I think of a pure uplifted face at a parsonage lattice in a Yorkshire wilderness; God and the awful stars above the graves of buried loves beneath, and all about the ineffable haunting witchery of the loud-whispering thorns. Certain it is we shall never have anything like the Brontës again until genius mates with like innocence and like loveliness—such intensity of genius yoked with such immensity of loveliness in the virgin forest of innocence." Of Miss Austen he adds, "Miss Austen's star arose at the close of a dull night in a gray morning presaging a clear day. . . . She is the Messonier of literature, art, and the fair mistress of its subtlest intricacies." Her own amiable personality, we read also, was reflected in her characters. "There was scarcely a charm in her most delightful characters that was not a true reflection of her own sweet temper and loving heart." All admirers of the three gifted Englishwomen should read Mr. Bonnell's "Studies."

* "Charlotte Brontë, George Eliot, Jane Austen." By H. H. Bonnell. (Longmans. 6s.)

### II.—DANNY: THE STORY OF A DANDIE DINMONT.*

"DANNY" is a dog story, and one which will delight dog-lovers, who find in the silent, sympathetic companionship of a dumb animal more solace, on occasions, than in that of the most demonstrative human being. There is, perhaps, a little too much doggy talk between Danny's mistress and himself, but that is easily forgiven to such a fascinating little vision, whose elusive charm Mr. Ollivant suggests rather than expresses. A quaint Scotch humour, lurking even in the pathos which marks, more or less, every scene in the book, gives to it a character all its own.

The old laird and his girl-wife, the grim serving-woman, Deborah Awe, and Robin Crabbe, the henchman, go to the making of the story of Danny and his doings. Very unpacific relations existed between Deborah and Robin, whom she affirmed was "a rude old man, the rudest old man in Hepburn save his Honour." But Danny loved him, and he loved Danny; yet though he loved Danny much, he feared the Laird more, for did not the Laird know full well all Robin's failings—"that he was vain-glorious above all men, that he dreamed dreams, that he drank whisky drams, and had a weeping eye."

This is how the girl-wife came into the life of his Honour. "Taciturn always, with the solitary humour of his race, the Laird had been sinking fast into that dark loneliness which seemed the habitual end of all members of his house, when she had come to him with innocent little ways, eyes of love, child laughter, to ripple through that house of silence and of shadow, like a sunlit wave. Missie had been his ward. Left derelict on the sea of life, motherless, brotherless, penniless, he had taken her, because, he could not help himself, into his house, and later for the same reason into his heart. She who had come to be his daughter stayed to be his wife; and he had loved her as some rough-handed labourer loves a tender lily of the fields." But his girl-wife was not spared long to him. Just as her soft influence was humanising and breaking down the barriers of isolation and reserve in which the Laird had entrenched himself, just as she was "fast winning him back to the ways of men and was emerging into light leading her old man by the hand—the sword fell." At first he ignored Danny's overtures, in which consolation was sought and refused. "He fenced himself round with an eternal loneliness. . . . He sought no sympathy and accepted none; but there was mute Danny thrusting on him no ill-timed condolences, yet urgent to be friends. He walled himself about with iron; but there was mute Danny, with importunate paw, craving admission." So the two began their silent lives in the naked house on the face of the moor. Then, by and by, the old Laird is unable to resist Danny's overtures; he had cherished him from the first for the sake of his lost wife; "later he bore with him because of his importunity, and at last he loved him for himself." There is much good character drawing besides that of Danny. Mr. Ollivant writes feelingly and vividly, whether it is of persons or scenery. How weirdly realistic are the last lines of the book. "In the old kirk, at the trysting place of the hills, sleeps the last of the Lairds of Hepburn, by the side of Missie and his grim forbears. Not far from the two he loved rests Danny. . . . Far away shines Burnwater like a jewel set beneath the established hills, and beyond the sea flashes like sheaves of shaken spears. . . . The curlews haunt the sky above him; the feet of fox, and the old grey brock, and all the enemies he loved, pass and repass over his grave and do not wake him; nor shall cold of snow nor heat of sun nor drumming wind, nor rain upon him, ever rouse him now. Warden of the marshes he keeps his watch beneath the stars faithfully for evermore."

* "Danny: The Story of a Dandie Dinmont." By Alfred Ollivant. (John Murray. 6s.)

### For Reading to the Sick.

"THE TENDER VOICE THAT CALLS."

A dimness of a glory glimmers here
Thro' veils and distance from the space remote
A faintest far vibration of a note
Reaches to us and seems to bring us near;
Causing our face to glow with braver cheer,
Making the serried mist to stand afloat,
Subduing languor with an antidote.
And strengthening love almost to cast out fear:
Till for one moment golden city walls
Rise looming on us, golden walls of home,
Light of our eyes until the darkness falls;
Then thro' the outer darkness burdensome
I hear again the tender voice that calls,
"Follow me hither, follow, rise, and come."

*Christina Rossetti.*

Life may become to us so favoured, so interesting, so precious if we take it all as the theatre on which we display before the eyes of God the glory of that hidden name which we have received from Him. That which we are in God's thought and intention, that is what we are discovering to ourselves and others at each passing hour. Let us ask ourselves, What is my name? What is the peculiar combination of moral qualities which is in us and no others? The seed cast into me of God—oh, that I knew what mystery was hidden in its silent history! Let the rains of God come, and the winds and the clouds pass over me, if only this name may break out and open into shape of flower and fulness of fruit, and so my name may be written broad and clear on my forehead, and all men may see it, and say, "He is not his own, he is God's. Behold the seal is on him. He is in the image of his Father. He is of the family of Christ."

Love is life and lovelessness is death. . . . Love, true pure love towards God and man, is the very essence and meaning of the Christian life. As the grace of God changes a man's heart and cleanses and sanctifies him, this is the great evidence of that change, this is the great difference which it makes—that he begins to grow in love, to lay aside self-seeking and to live for others—and so he may know that he has passed from death unto life.

*Canon Scott-Holland.*

He who patiently and meekly bears the difficulties disappointments, anxieties, rebuffs, vexations, irritations, troubles, and heartaches which beset the daily path of life in which God has placed us, including those which we often raise for ourselves in our hidden life, saying, "It is my due," uniting his cross to that of his Master, will find pain and bitterness changed to sweetness and rest at last, and will for ever give thanks for what once seemed the troubles which marred and frustrated his ease and happiness.

*S. Lear.*

O Lord, my heart is ever prone
To seek the things I hear and see,
To live to this vain world alone,
As though the words were all unknown
"Take up thy cross, and follow Me."

Who truly bear the Christian name
Must walk *as* Christ—one never free
From pain and peril, grief and shame;
His pattern sealing this His claim,
"Take up thy cross, and follow Me."

*T. Judkin.*

### Notes and Queries.

FOR REGULATIONS SEE PAGE 195.

*Home.*

(151) Will you kindly inform me where a man of 65, suffering from epilepsy, could be received upon payment of not more than £1 per week?—*E. M.*

The Home for Epileptics, Maghull, near Liverpool (office address), the Hon. Secretary, 2 Exchange Street East, Liverpool.

*West Africa.*

(152) Can you tell me to whom I should apply for an appointment in a hospital in West Africa?—*H.*

The Secretary, the Colonial Nursing Association, Imperial Institute, S.W.

*Maternity.*

(153) I have taken the Liverpool certificate and am going in for the L.O.S., but as I am doing this with a view to directing a charitable maternity home to be opened shortly, I should like to gain as much experience as possible. Do you know of any institution where I could get this experience in return for my services without salary?—*A. B. C.*

You might possibly get what you want by advertising.

*Male Nurse.*

(154) Will you tell me what steps to take in order to become a male nurse; also in order to become a male attendant?—*Anxious.*

Train at the National Hospital for the Paralysed, Queen Square, Bloomsbury, W.C., and in an Asylum for the Insane to become a male attendant.

*Nursing in Edinburgh.*

(155) Will you kindly tell me how I can hear of vacancies in a private institution in Edinburgh; and will you also give me the address of the co-operation for private nurses?—*E. P.*

We do not give the names of private nursing institutions. The Royal Scottish Nursing Association, 69 Queen Street, Edinburgh, is a large association managed by a committee which offers very fair terms and advantages. Write to the Lady Superintendent.

*L.O.S.*

(156) I shall be pleased if you will tell me if there are any classes which I could attend for a course of instruction for the L.O.S. examination.—*Becky Sharp.*

Write to the Secretary, the Midwives' Institute, 12 Buckingham Street, Strand, W.C.

*Surgical Work.*

(157) Will you kindly advise me as to the best book on surgical work of a simple kind? I should like it up to date as I am the district nurse of a large village.—*Nurse M.*

Possibly "Surgical Ward-work and Nursing" would suit you. It is by Alexander Miles, M.D.Edin., C.M., F.R.C.S.Eng., and can be obtained, price 3s. 10d. post free, from the Scientific Press, 28 & 29 Southampton Street, Strand, W.C.

*Massage. Dispensing.*

(158) Will you please tell me where I can get the best and cheapest lessons in massage; also, where I could have lessons in dispensing, and what the cost would be?—*Sister Dora.*

Write to the Secretary the Incorporated Society of Trained Masseuses, 12 Buckingham Street, Strand, and also to the Secretary of the Pharmaceutical Society, 17 Bloomsbury Square, W.C.

*Indian Nursing Service.*

(159) I should be very much obliged if you will kindly let me know to whom I should apply for the particulars and form of application of the Indian Nursing Service.—*J. S. G.*

Apply to the India Office, St. James's Park, S.W.

*Caul.*

(160) *Nurse F.* would be much obliged if you will tell her the best paper in which to advertise a caul, as she has one to dispose of.

Any medical paper would serve your purpose.

**Standard Nursing Manuals.**

"The Nursing Profession: How and Where to Train." 2s. net; 2s. 4d. post free.

"Nursing: Its Theory and Practice." (Revised Edition). 3s. 6d. post free.

"Surgical Ward Work and Nursing." (Revised Edition). 3s. 6d. net.; 3s. 10d. post free.

"Practical Handbook of Midwifery." (New Edition). 6s. net; 6s. 3d. post free.

"Notes on Pharmacy and Dispensing for Nurses." 1s. post free.

"Fevers and Infectious Diseases." 1s. post free.

"The Art of Massage." (New Edition). 6s. post free.

